# Correction: Weng, J., et al. DNA Methylation Analysis Identifies Patterns in Progressive Glioma Grades to Predict Patient Survival. *Int. J. Mol. Sci.* 2021, *22*, 1020

**DOI:** 10.3390/ijms22083842

**Published:** 2021-04-08

**Authors:** Jingyin Weng, Nicole Salazar

**Affiliations:** Department of Biology, San Francisco State University, San Francisco, CA 94132, USA; jweng2@mail.sfsu.edu

The authors wish to make the following corrections to paper “DNA Methylation Analysis Identifies Patterns in Progressive Glioma Grades to Predict Patient Survival” [1]:

In Figure 4C, the *p*-value for the *Fn14* (cg00510447) box plot is incorrect. Figure 4 should be replaced with the following figure (Figure 1).

In Figure 6, the methylation status requirement shown in the schematic diagram is reversed but the subsequent analysis is not impacted. Figure 6 should be replaced with the following figure (Figure 2).

In the main text, there was an error in Paragraph 1, 3rd sentence of Section 2.1. The definition of hypo- and hypermethylated CpG sites outputted from the differential variability analysis is an inexact description. A correction has been made to this as presented below:

In the comparison between Grade II and Grade III gliomas, 2095 and 146 CpG sites (1.54 × 10^−10^ ≤ *p*-value ≤ 0.00062, 2.75 × 10^−5^ ≤ false discovery rate (FDR) ≤ 0.05) were methylated at a consistent level in one glioma grade, while the other glioma grade shows hypo- or hypermethylation variability (Figure 1A). 

The authors apologize for any inconvenience caused and state that the scientific conclusions are unaffected. The original article has been updated.

## Figures and Tables

**Figure 1:**
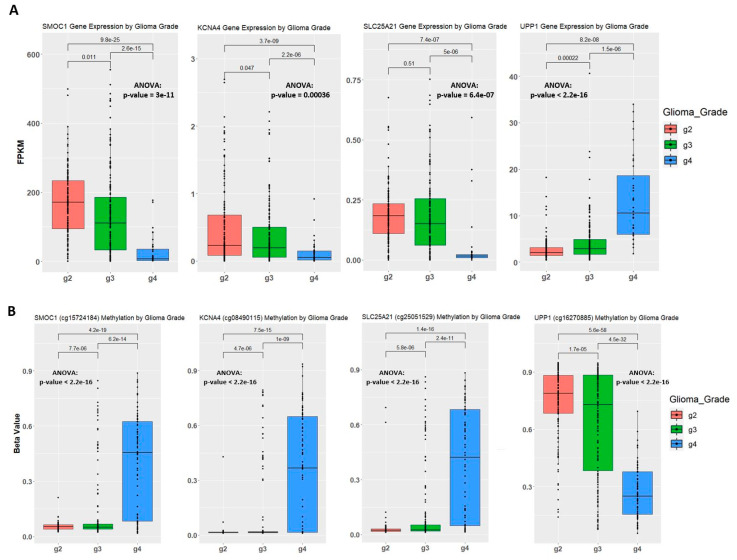

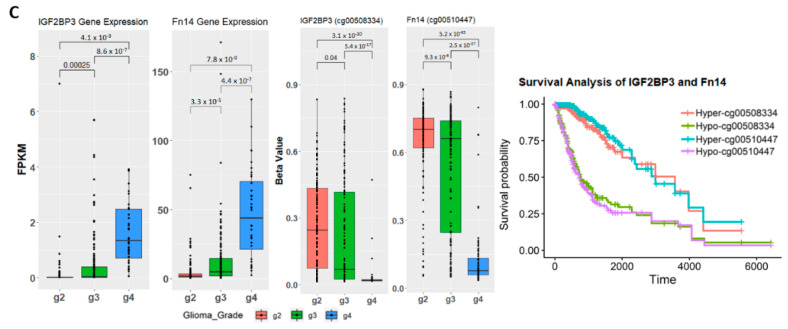
Correlation between gene expression and methylation level in the training set. (**A**) Gene expression of samples by glioma grade. Fragments Per Kilobase Million (FPKM) stands for Fragments Per Kilobase of transcript per Million mapped reads. The t.test method is used for statistical analysis when comparing two groups of samples. One-way analysis of variance (ANOVA) was used when comparing three groups of samples on one variable. (**B**) Methylation level of samples by glioma grade. Methylation level is represented by beta-value. One probe is selected to show the methylation level for *SMOC1* gene and *KCNA4* gene. (**C**) Boxplots of *IGF2BP3* and *Fn14* gene expression and methylation level on one of the associated probes. Kaplan-Meier plot measuring patient survival through the methylation level of CpG sites captured by cg00508334 for *IFG2BP3* and cg00510447 for *Fn14*.

**Figure 2:**
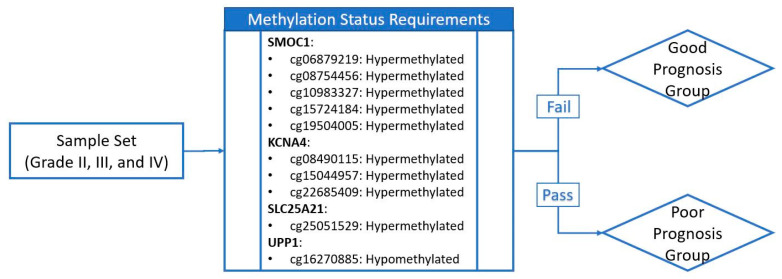
Schematic diagram of sample grouping criteria. If the sample meets methylation status requirements for all ten CpG sites (represented by probe IDs from the array product design), it is assigned to the poor prognosis group. If the sample fails one or more of the methylation status requirements, it is assigned to the good prognosis group. Hypo- or hypermethylation status of the sample is determined through comparison with the group median methylation level.
